# Evaluating the expression level of HERV-K env, np9, rec and gag in breast tissue

**DOI:** 10.1186/s13027-019-0260-7

**Published:** 2019-11-29

**Authors:** Shaian Tavakolian, Hossein Goudarzi, Ebrahim Faghihloo

**Affiliations:** Department of Microbiology, School of Medicine, Shahid Beheshti University of Medical Sciences, Tehran, Iran

**Keywords:** *Env*, *np9*, *Rec*, *Gag*, Breast cancer

## Abstract

**Objective:**

Breast cancer is one of the most common health problems. It has been suggested that several risk factors, either considered as external or internal, play a critical role in the pathogenesis of breast cancer, which among them, HERV-k, has the most fundamental role. In the present study, we aimed to evaluate the role of HERV-k *env, gag, rec, np9* expressions in breast cancer progression.

**Materials and methods:**

We collected 40 breast cancer tissues and their normal adjacent ones. After extracting the RNA of breast samples, we evaluated the expression of HERV-k *env, gag, rec, np9* by using Quantitative real-time PCR (qRT-PCR).

**Results:**

The resulting data revealed that while there was a meaningful increase in the expression level of HERV-k *env, gag and np9* in breast cancer tissues (*P* ≤ 0.01, 0.05, 0.05, respectively), we failed to find any significant elevation in the expression level of *rec* mRNA level.

**Conclusion:**

The results of our study suggested that there is a plausible correlation between the mRNA expression level of HERV-K *env, gag* and *np9* and the progression of breast cancer, proposing these markers as promising biomarkers to diagnose breast cancer.

## Introduction

The diagnostic approaches in breast cancer, as the second most prevalent cause of cancer death in womankind [1, 2], still remain challenging for clinician, opening the way for the recognition of new biomarkers for early detection of this malignancy. The heterogeneous characteristic, complex etiology, diverse genetic mutation and the various distinct clinical manifestations suggested that a wide variety of internal and external risk factors could be involved in the pathogenesis of breast cancer. Apart from external risk factors, such as obesity, smoking, consumption of alcohol and the level of melatonin hormone, internal risk factors, including genetic and epigenetic could initiate signaling pathways in breast cancer through regulation of different genes [3–9].

Previous studies have declared that Human endogenous retrovirus (HERV) is one of the demanding factors in cancer progression, since this virus could stimulate neoplastic cells to proliferate continuously and to evade from apoptosis [10]. Upon 30–40 million years ago, since the first time that HERV has entered into the germ line cells, the genes of this virus has transmitted vertically into the host’s genome [11], comprised approximately 8% of human genes. According to exogenous division, instead of phylogenetic one, HERV is classified into three classes, including class I (gammaretrovirus- and epsilonretrovirus-like), class II (betaretrovirus-like) and class III (spumaretrovirus-like) [12–15]. Among all HERV families, HERV-K has integrated into cell genome more recently. Given to its set of intact open reading frames and biological activity, this sub-family of HERV could synthetize some retrovirus-like particles [16]. Moreover, it has been reported that the long terminal repeats (LTR) sequence in HERV regulates the expression of structural genes, including HERV-K *np9, rec, env* and *gag* genes [17], which could initiate autoimmune diseases and different types of cancers [18]. As the relationship between the expression of HERV-K *np9, rec, env and gag* and breast cancer remains to be an open to debate, in this study, we evaluated the expressions of HERV-K *np9, rec, env* and *gag* genes in patients suffering from breast cancer.

### Methods

#### Samples

This study was approved by the Shahid Beheshti University of Medical Sciences” IR.SBMU.MSP.REC.1398.563 (Grant no 17631). For evaluating the expression level of HERV-k *env, gag, rec, np9*, 40 breast cancer (76.4, mean age) and their adjacent normal breast tissues, which was at least 5 cm away from tumor, have been collected from Emam hossein and Taleghany hospital between 2018 and 2019 in Tehran, Iran. All tissues were stored in RNA latter (Qiagen GmbH, Hilden,Germany) in − 20^°C^. The stage of malignant tissues was confirmed by expert pathologists and the information of patients is summarized in Table 1. The samples of those patients who were treated either by chemotherapy or radiation were excluded from this study.

**Table 1:** The Clinical characteristics of 40 breast cancer patients

> 60	69%
Tumor localization	(Left: 45%) - (right: 55%)
Family history	(Absent: 88.1%) - (Present: 11.9%)
Lymph node metastasis	(Negative: 48%) - (positive: 52%)
Tumor size	(> 2 cm: 62.8%) - (< 2 cm: 37.2%)
Tumor stage	(I-II: 48%) - (III-IV: 52%)
Estrogen receptor (ER) status	(Negative: 54.3%) – (positive: 45.7%)
Progesterone receptor (PR) status	(Negative: 46.6%) – (positive: 53.4%)

### RNA extraction

All tissues were suspended in 1 ml RNX-plus solution reagent (Cinnagen, Tehran, Iran) in homogenizer. After adding chloroform and centrifuging, protein was extracted from solution. Afterwards, we added isopropanol to the solution to precipitate the RNA of supernatant. Finally, RNA plate was diluted with 50 ul of DEPC-treated water. To confirm the purity of RNA, all of RNA samples were run in agarose gel electrophoresis in order to observe 5S, 18S and 28S bands. (To prevent the role of DNA, we used DNAse.)

### cDNA synthesis

The reverse transcription reaction was performed using Bio fact cDNA kit (Daejeon, South Korea). To convert RNA into DNA, we provided a 20 μl reaction containing 1 ul random hexamer, 9 ul master mixes and 10 ul of RNA samples. The incubation was carried out for 40 min at 50 ^°C^ and 10 min at 95 ^°C^ in Bio Intellectica PCR. The synthesized cDNAs were then diluted twofold in sterile water.

### Quantitative real-time PCR

Alterations in the expression level of mRNAs were assessed using Real-time RCR analysis. We have combined 10 ul BIOFACT™ 2X real-time PCR master mix (for SYBR Green I; BIOFACT, South Korea), 6 ul sterile water, 1 ul forward 10 pmol, 1ul reverse primer 10 pmol,2 ul cDNA in a final 20 ul volume, and incubated in one cycle at 95^°C^ for 10 min, 40 cycles at 95^°C^ for 30s; 55 ^°C^ for 30s, 72^°C^ for 30s in Rotor-gene 6000 (Corbett life sciences, Sydney, Australia) in 36-well Gene Discs. The melt curve was between 60^°C^ and 95 ^°C^. GAPDH housekeeping gene was amplified as an internal control, and the values for the relative quantification were calculated based on 2^– ΔΔct^ expression formula. (For every gene, we used a negative control to demonstrate that there was no cross contamination between samples, in real-time PCR.)The list of primers used in this study is summarized in Table 2. (Alignment with BioEdit software was performed to avoid cross reaction of our primers used in this study with complete MMTV genome.)

**Table 2:** Nucleotide sequences of primers used for real-time RT-PCR

Gene	Forward primer (5′-3′)	Reverse primer (5′-3′)
GAPDH	ATGTTCGTCATGGGTGTGAA	GGTGCTAAGCAGTTGGTGGT
rec	ATCGAGCACCGTTGACTCACAAGA	GGTACACCTGCAGACACCATTGAT
np9	AGATGTCTGCAGGTGTACCCA	CTCTTGCTTTTCCCCACATTTC
env	TAACCCTGTCACTTGGATT	ATGTCACTGTCTCTTCGG
gag	AGCAGGTCAGGTGACCGTAAC	GGTGCCATAGCATTGTCTCCT

### Statistical analysis

To analyze the results of mRNA expression, we used Graph-PadPrism software. Experimental data are expressed by mean ± standard deviation of three independent assays. Statistical significance was calculated using ANOVA tests. *P*-value less than (*P* < 0.05) was used for the differences.

### Results

#### Samples

To evaluate the expression level of different HERV-K associated genes in mammary carcinoma, 40 breast cancer tissues and their normal adjacent parts were collected from Emam hossein and Taleghany hospital. All tested tissues were analyzed by two expert pathologists and it has been confirmed that more than 50% of patients possessed a tumor with stage of III-IV (Table 1).

### Evaluation in the expression level of HERV-K associated genes in breast cancer tissue

**Fig. 1:**
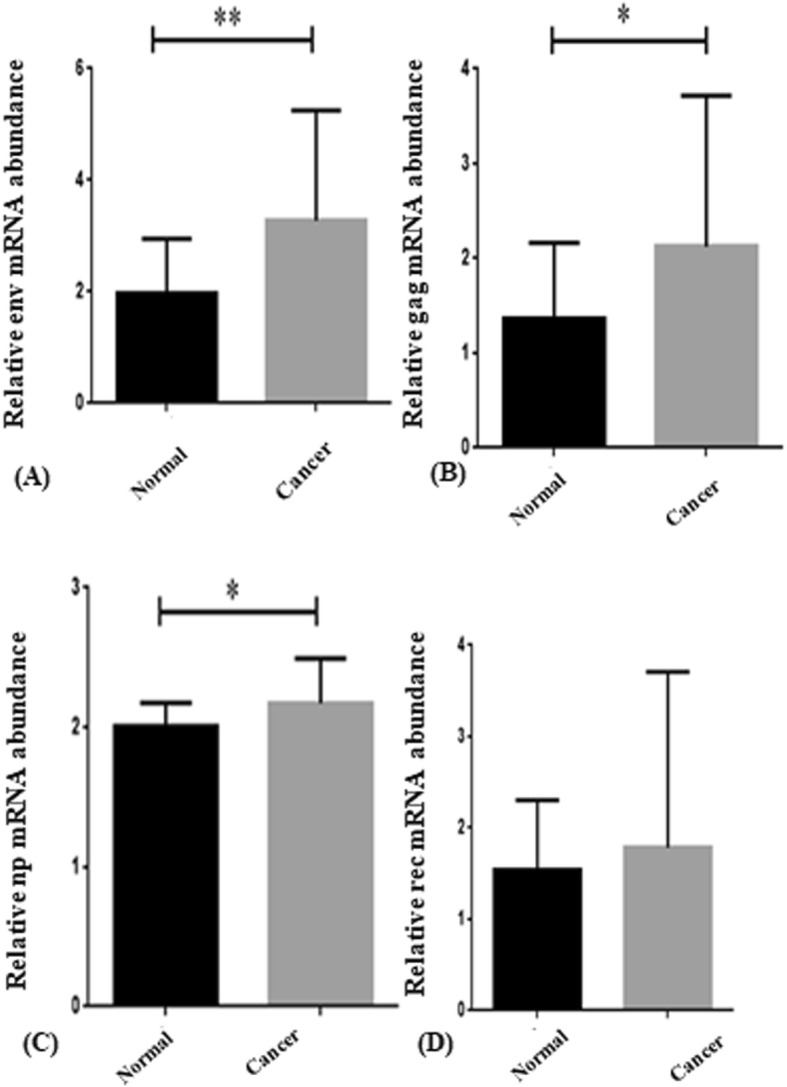
**a.** *env* mRNA expression in the breast cancer compared to normal tissues. The expression level of *env* remarkably increased in the breast cancer tissues as compared to normal cells. ** *P* ≤ 0.01 represents significant changes from normal tissue. **b.** *gag* mRNA expression in the breast cancer tissues compared to normal tissues. The expression level of *gag* increased significantly in breast cancer tissue. * *P* ≤ 0.05 represents significant changes from untreated control. **c.** Evaluating the mRNA expression level of *np9* in normal and breast cancer tissue. The results of RQ-PCR analysis revealed that there is a significant elevation in the expression of *np9* in breast cancer tissues as compared to normal counterparts. * P ≤ 0.05 represents significant changes from normal tissue. (Values are given as mean ± standard deviation of three independent experiments). **d** The comparison between the mRNA expression level of *rec* in the normal and breast cancer tissues

To assess whether there was a correlation between the expression level of LTR-regulated genes and the progression of breast cancer, we extracted RNA from the tissues. RQ-PCR analysis revealed that there was a significant elevation in the mRNA level of *env* in comparison with the normal tissue (** *P* value < 0.01) (Fig. 1. A).

Moreover, our results showed that while the ranscription level of *np9* and *gag* increased in breast cancer tissue, this expression remained unchanged in normal adjacent tissues, highlighting that probably there is an association between the expression of these genes and the progression of breast cancer (*P value < 0.05) (Fig. 1. B, C).

Unlike the expression of aforementioned genes, we could not find any significant alteration in the expression level of *rec* either in breast cancer tissue, or in normal adjacent parts (Fig. 1. D).

## Discussion

Mounting body of evidence has enumerated human endogenous retrovirus (HERV) families can be considered as one of the most important risk factors in the progression of human cancers, especially in breast cancer [10, 19]. Among different sub-groups of HERV, the results of some studies has declared that Herv-k *env, gag, rec,* and *np9* genes could stimulate the invasive properties of cancer cells, giving them an opportunity to expand to other tissues and areas. Furthermore, the mouse mammary tumor virus (MMTV) as another risk factor, which is belonged to genus *Betaretrovirus* in cancer progression, cannot be ignored. However, the exact role of these viruses in the progression of breast cancer remains to be an open to debate. In the present study, we aimed to evaluate whether there was a correlation between the expression of HERV-K *env, gag, rec, np9* and the progression of breast cancer.

The association between the expression of *env* gene and the progression of breast cancer has emerged from the recent disclosure indicating that HERV-K and MMTV *env* gene could initiate the progression of breast tumor through modulation of Ras/Raf/MEK/ERK signaling pathway [20]. The results of the present study showed that *env* gene is over-expressed in breast cancer samples in comparison with the normal tissues. This finding was consistent with the study conducted by Johanning, et al. who showed that the expression of *env* increased not only in various malignant breast cell line (MDA-MB-231, MCF-7, SKBR3, MDA-MB-453, T47D, and ZR-75-1) compare to non-malignant ones (MCF-10A and MCF-10AT) [21], but also elevated during breast cancer [22]. The correlation between *env* and breast cancer has also been reported in several other studies [23, 24]. Moreover, in another study, it has been reported that the expression of *env* gene increased in invasive ductal carcinoma as compare to normal ones. Zhao et al. also delineated that both Chinese and American patients who had breast cancer displayed an over-expressed *env*; however, the expression of this gene was normal in healthy women [25]. Naccarato AG and et al. shown, exogenous MMTV *env*-like sequences (MMTVels) was presented higher in sporadic breast cancer in comparison with hereditary breast cancer; therefore this virus can be related with sporadic breast cancer [26]. Nevertheless, in some countries, such as Saudi Arabia, the sequences of MMTVels was reported low frequency [27]. Recently, Salmons B and et al. wrote a review article in relationship between MMTV and human breast cancer, and also studied on different MMTV mechanisms of oncogenesis, and they suggested further studies may confirm the effect of this virus on breast cancer invasion [28].

Another HERV-associated gene whose its expression was elevated in malignant tissues was HERV-k *np9*. There are several reports claiming that the expression level of this gene is increased in a variety of human cancers, ranging from solid tumors, such as breast cancer [25, 29] and melanoma [30], to chronic hematologic malignancies [31]. The oncogenic property of *np9* is also evident in transformed cell lines, where it has been reported that this gene could serve as a growth factor for cancer cells [32]. The association between *np*9 gene and several signaling pathways, including WNT, ERK, Akt and Notch1 suggested that probably the over-expression of this gene could play a crucial role in the progression of breast cancer [33, 34]. Apart from *np*9, we also found that there is a significant over-expression in the mRNA level of *gag*. Interestingly, the relationship between *gag* expression and the incidence of human cancers has been evaluated in different recent studies; however, in many cases there are conflicting results. While a previous report conducted by Gary L. Johanning could not find any changes in the expression level of *gag* between breast cancer tissues and normal counterparts [22], other study reported that the patients with breast cancer possessed the higher mRNA level of *gag* [35]. The association between *gag* and human cancer is not restricted in breast cancer and the expression of this gene is considered as a biomarker for prostate cancer [36]. The other gene which its expression is regulated by LTR is HERV-k *rec*. Although the inhibitory effect of this gene on apoptosis has been reported in several human cancers, including skin [37] and ovarian cancer [38], we failed to find any association between this gene and the incidence of breast cancer, which may be due to high variation of expression levels in cancer tissues; moreover, mismatches may have an effect on the expression of *rec*. Klein D indicated minor mismatch, including point mutations in the primer or the probe region can lead to decrease in the quantification; conversely, major mismatches of three or four nucleotides can inhibit the real-time PCR detection, completely [39].

## Conclusion

The results of the current study suggested that the expression level of HERV-K *env*, *gag* and *np9* was elevated in breast cancer cells; introducing these genes as a promising biomarkers for the early diagnosis of breast cancer.

## Data Availability

Please contact author for data requests.
